# Feasibility of contrast-enhanced ultrasound and flank position during percutaneous nephrolithotomy in patients with no apparent hydronephrosis: a randomized controlled trial

**DOI:** 10.1007/s00345-022-03933-4

**Published:** 2022-01-21

**Authors:** Zeng-Qin Liu, Jing Xie, Chu-Biao Zhao, Yan-Feng Liu, Zai-Shang Li, Ji-Nan Guo, Hong-Tao Jiang, Ke-Feng Xiao

**Affiliations:** 1 Department of Urology, Shenzhen People’s Hospital (The Second Clinical Medical College, Jinan University; The First Affiliated Hospital, Southern University of Science and Technology), Shenzhen, 518020 Guangdong China; 2 Shenzhen Engineering and Technology Center of Minimally Invasive Urology, Shenzhen People’s Hospital, Shenzhen, 518020 Guangdong China

**Keywords:** Contrast-enhanced ultrasound, Flank position, Percutaneous nephrolithotomy, Calyceal fornix, Kidney stone

## Abstract

**Purpose:**

To investigate the puncture accuracy and feasibility of contrast-enhanced ultrasound (CEUS) guided percutaneous nephrolithotomy (PCNL) in flank position for patients with no apparent hydronephrosis.

**Methods:**

Between May 2018 and June 2020, 72 kidney stone patients with no or mild hydronephrosis were randomized into two groups: a CEUS-guided PCNL group and a conventional ultrasound (US)-guided group. Patients’ demographics and perioperative outcomes were compared, including the success rate of puncture via calyceal fornix, the success rate of a single-needle puncture, puncture time, operative time, postoperative hemoglobin loss, stone-free rate, incidence of complications and postoperative stay.

**Results:**

The success rate of puncture via calyceal fornix for CEUS-guided group was significantly higher than that for conventional US-guided group (86.1 vs. 47.2%, *p* = 0.002). Patients performed with CEUS-guided PCNL required shorter renal puncture time than those guided with conventional US (36.5 s vs. 61.0 s, *p* < 0.001). The median postoperative hemoglobin loss in the CEUS-guided group was significantly lower than that in conventional US-guided group (2.5 vs. 14.5 g/L, *p* < 0.01). There was no statistically significant difference in the success rate of a single-needle puncture, operative time, stone-free rate, incidence of complications and postoperative stay between the two groups.

**Conclusion:**

CEUS guidance facilitates identification of the renal calyx fornix, and benefits more precise renal puncture and less hemoglobin loss in PCNL. CEUS-guided PCNL in flank position is a feasible approach to the treatment of kidney stone patients with no apparent hydronephrosis.

Trial registration number: ChiCTR1800015417.

**Supplementary Information:**

The online version contains supplementary material available at 10.1007/s00345-022-03933-4.

## Introduction

Percutaneous nephrolithotomy (PCNL) is recommended as the standard procedure for upper urinary tract stones larger than 2 cm [1]. Despite of the high stone clearance rate and many refinements of PCNL, there are still concerns regarding the potential risk of severe complications, including surrounding organ injury, bleeding requiring transfusion or embolization, or even death [2]. In a recent study, puncture correctness was found to be the only significant factor related to severe bleeding requiring angioembolization after PCNL [3]. Therefore, precise renal puncture is a critical step in PCNL procedure.

Fluoroscopic guidance, traditionally used for renal access, allows accurate identification of the targeted calyx for puncture. The main disadvantage of fluoroscopic guidance is lack of real-time visualization of adjacent viscera, which may increase the risk of surrounding structures injury. Moreover, its ionizing radiation exposure may have detrimental effects on exposed patients and medical personnel [4]. As an alternative imaging method, ultrasound (US) guidance has been proven to be effective and safe for PCNL and experienced increasing popularity worldwide [5, 6]. Its advantages include no radiation exposure, real-time monitoring of the collecting system, renal parenchyma and surrounding organs, detection of radiolucent stones and avoid vascular injury with Doppler flow imaging. However, US-guided PCNL remains challenging in patients with no apparent hydronephrosis, because it is difficult to visualize the targeted calyces suitable for puncture.

Previous studies have shown that intraoperative use of contrast-enhanced ultrasound (CEUS) in the prone position can achieve better visibility of nondilated collecting systems and facilitate more accurate puncture [7, 8]. However, the puncture accuracy and safety of CEUS-guided PCNL in flank position has not been investigated so far. The aim of the present study was to evaluate the effectiveness and safety of CEUS-guided PCNL in flank position for kidney stone patients with no apparent hydronephrosis, and compare it with conventional US-guided PCNL.

## Materials and methods

### Trial design

This prospective, single blind, 1:1 randomized controlled trial was performed at Shenzhen People’s Hospital according to the Declaration of Helsinki. The study protocol was approved by the Institutional Ethics Committee (LL KT 20170401104), and registered with the Chinese Clinical Trial Registry (ChiCTR1800015417).

### Participants

Patients with kidney stones requiring PCNL at our institution were assessed for eligibility. Stone size was defined as the longest axis on preoperative non-contrast computed tomography. Inclusion criteria were: (1) age 18–70 years; (2) demonstrated no or mild hydronephrosis on preoperative intravenous urography. Mild hydronephrosis was defined as slight distension of the renal pelvis or bluntness of the cup of renal calyx, with the papillae still easily identified. Exclusion criteria included the following: (1) pregnant patients; (2) solitary kidney (anatomical or functional); (3) acute urinary tract infection; (4) coagulation dysfunction; (5) severe cardiopulmonary insufficiencies; (6) patients with horseshoe kidney, polycystic kidney, or pelvic kidney; (7) morbid obesity (BMI > 40); (8) patients with abnormal renal function (serum creatinine > 1.2 times the upper limit of normal). After signing informed consents, the eligible patients were randomized into CEUS-guided PCNL group and conventional US-guided PCNL group, using computer-generated randomized table.

### Interventions

We used sulfur hexafluoride microbubbles (SonoVue; Bracco, Switzerland) as ultrasound contrast agent. First, 5 ml of physiologic saline was injected into a bottle of microbubbles, which was shaken for 20 s for sufficient mixture. Then, 1–2 ml of the mixture was injected into a 50 ml injection syringe containing 50 ml saline, and the preparation of ultrasound contrast agent was completed. Under general anesthesia and with the patient supine, an open-ended 6-F ureteral catheter was retrograde placed into the ipsilateral proximal ureter up to 25 cm under ureteroscopic guidance. Patients were then moved to a complete lateral decubitus position. We used a B-K Medical US machine (Pro Focus 2202, Herlev, Denmark) to guide PCNL, which allowed B-mode and contrast-enhanced mode imaging to be displayed simultaneously on one screen. For CEUS-guided PCNL, we slowly injected the contrast agent via the preplaced ureteral catheter until the entire collecting system was delineated. Generally, 50–100 ml of contrast agent was injected for each case. Then an 18-gauge needle (Create Medic, Yokohama, Japan) was inserted towards the brightest spot of the targeted calyx. After removing the needle core, we could see a bright contrast outflow tract under the contrast-enhanced mode imaging (Supplementary Fig. 1), or efflux of urine through the puncture needle, which confirmed entry into the collecting system. Percutaneous tract dilation and the remainder of the procedure were performed routinely. For conventional US-guided PCNL, physiologic saline was retrograde injected to dilate the collecting system, and other procedures were the same as CEUS-guided PCNL. All PCNLs were performed solely under US guidance and through one tract with size of 18 Fr, by two urologists (Z.L. and K.X.) with at least 200 cases experiences of US-guided PCNLs. Prior to starting this study, both urologists had successfully performed 5 cases of CEUS-guided PCNL and mastered this technique.

### Outcomes

The primary outcome was the success rate of puncture via calyceal fornix, confirmed by intraoperative findings and anterograde pyelography through nephrostomy tube performed 3 days after surgery (Supplementary Fig. 2). Secondary outcomes included (1) the successful rate of single-needle puncture, defined as one-shot needle puncture confirmed by contrast-enhanced mode imaging or efflux of urine through the puncture needle, (2) renal puncture time, defined as the time from inserting a needle through the skin to successful entry into the collecting system, (3) stone-free rate, defined as residues of 4 mm or smaller on non-contrast CT 4 weeks after surgery. Operative time, hemoglobin loss, postoperative hospital stay and complications were also noted.

### Sample size

Our hypothesis was that CEUS guidance had superior effect on renal puncture compared with conventional US guidance, with a higher success rate of puncture via calyceal fornix (80 vs. 40%, respectively). Using a two-sided chi-squared test with a power of 90% and a significance level of *α* = 0.05, a sample size of 60 was required, with 30 patients each group (CEUS and conventional US-guided PCNL). Considering a drop-out rate of 20%, 72 patients were enrolled in the trial.

### Statistical analyses

Statistical analyses were performed using SPSS (version 25). Normally distributed continuous data were shown as mean (SD) and analyzed by Student’s *t* test. Non-normal data were expressed as median (quartile 1, quartile3), and analyzed by Mann–Whitney *U* test. Categorical variables, shown as the number and/or percentages, were analyzed by the chi-square test or the Fisher exact test. Data were analyzed at 95% CI and *p* < 0.05 was considered statistically significant.

## Results

Between May 2018 and June 2020, a total of 72 patients were included in this study. Among these patients, 36 were randomized to CEUS-guided group and 36 to conventional US-guided group. No patient withdrew during the treatment. Demographic and stone characteristics of the patients were comparable between the two groups (Supplementary table 1).

Renal punctures were successfully performed in all patients under the guidance of CEUS, while failed in three patients under conventional US guidance and fluoroscopy was adopted. The success rate of puncture via calyceal fornix for CEUS-guided group was significantly higher than that for conventional US-guided group (86.1 vs. 47.2%, *p* = 0.002, Supplementary table 2). The median puncture time for CEUS-guided PCNL was also found to be shorter than those for conventional US (36.5 vs. 61.0 s, *p* < 0.001). In addition, the median postoperative hemoglobin loss in the CEUS-guided group was significantly lower than that in conventional US-guided group (2.5 vs. 14.5 g/L, *p* < 0.01). The two groups showed no significant difference with regarding to the success rate of a single-needle puncture, the stone clearance rate, operative time and postoperative stay.

The overall complications were evaluated using the Clavien-Dindo grading system [9]. It was found to be comparable between the two groups regarding the overall perioperative complication rate (16.7 vs. 13.9%, *p* = 0.11). However, we found CEUS-guided PCNL was associated with less need of blood transfusion (0 vs. 8.3%), although there was no statistical significance. No adverse events, such as headache, nausea, vomiting and dizziness, were observed associated with retrograde ultrasound contrast injection.

## Discussion

The present study demonstrated that CEUS-guided PCNL in flank position is feasible and safe for kidney stone patients with no apparent hydronephrosis. Our results showed that CEUS-guided PCNL achieved significant higher success rate of puncture via calyceal fornix, shorter renal puncture time and less postoperative hemoglobin loss than conventional US-guided PCNL in patients with no or mild dilated collecting systems. The success rate of a single-needle puncture, stone clearance rate and need of blood transfusion in CEUS-guided group were also superior to that of conventional US-guided group, although there was no statistical significance.

Establishing optimal access to the pelvicalyceal system is the most challenging and crucial step of a successful PCNL. The acknowledged ideal location for renal puncture is through cup of the renal calyx, which is associated with minimal vascular injury and offer optimal access to stone clearance. In the presence of hydronephrosis, the renal calyx fornix is easy to distinguish since it demonstrates as a hyperechoic area adjacent to the hypoechoic urinary space. However, it is difficult to identify this structure in nondilated collecting system under US guidance, owing to the poor imaging affected by the peripelvic fat. Thus, puncture in nondilated collecting system under US guidance remains a challenging scenario for urologists. In the previous literature, the success rate of US-guided PCNL was 96–100% in patients with obstructed dilated system, but decreased to 82% in patients with a nondilated collecting system [10]. Study has also showed that patients without hydronephrosis were associated with significant lower stone-free rate and longer operation time [11]. Moreover, in the absence of hydronephrosis, repeated punctures may be necessary to access the desired calyx, which significantly increase the risk of vascular injury in PCNL. Multivariate logistic regression analysis showed that absence of hydronephrosis was a significant risk factor for blood transfusion in conventional PCNL [11].

Retrograde saline injection was traditionally performed by many surgeons to distend the collecting system and improve the visibility of the targeted calices. However, this method may be associated with high intrapelvic pressure and increase the risk of perioperative infections [12]. Intravenous injection of furosemide was also reported to increase the degree of collecting system dilatation and facilitate effective renal access [13]. The disadvantage of this approach was the potential risk of medication side effects such as acute renal insufficiency [14]. Other techniques have also been reported to access the collecting system, including ureteroscopic-assisted percutaneous access, “all-seeing needle” and automated needle targeting with X-ray-Robot-assisted device [15–17]. However, these innovations require additional equipment and might pose challenges with regards to convenience and cost.

In 1982, Armstrong and colleagues first reported the clinical application of CEUS [18]. Since then, this technique has been widely applicated in intracavitary administration [19]. Its safety for intracavitary administration has been proved by numerous clinical studies and minor adverse effects have been reported in < 0.5% of patients [20]. Chi et al. first introduced CEUS to aid US-guided PCNL in five patients without hydronephrosis, via retrograde ultrasound contrast injection to improve the visibility of the collecting system, and achieved high puncture success rate up to 100% [7]. Guo and colleagues demonstrated that compared to conventional US-guided PCNL, CEUS-guided PCNL achieved higher successful rate of one puncture, shorter puncture time and lower postoperative hemoglobin loss [8]. Subsequently, Xia and coworkers conducted a prospective, randomized trial in 154 kidney stone patients with nondilated collecting system, to investigated the efficacy and safety of CEUS-guided PCNL [21]. They concluded that CEUS-guided PCNL outperformed conventional US-guided PCNL, with a higher success rate of a single-needle puncture, less needle passes, shorter puncture time, and lower postoperative hemoglobin drop. It is worth noting that these aforementioned studies only evaluated the efficacy of CEUS-guided PCNL in prone position. The renal puncture accuracy has not been well assessed, and the clinical value of CEUS-guided PCNL in flank position remains unclear.

This study evaluated the success rate of puncture via calyceal fornix in participants by postoperative anterograde pyelography, which was seldom reported in the previous literature. Our data showed the CEUS-guided PCNL achieved more precise puncture in patients with no or mild hydronephrosis, with a rate of puncture via calyceal fornix up to 86.1%. Recently, studies have showed that nonpapillary or infundibular puncture was not associated with higher blood loss or transfusion rate compared to calyceal puncture [22, 23]. However, these reports are from single center and have not been recognized worldwide. Currently, papillary puncture is still the acknowledged standard approach in PCNL. In the present study, we found it was not reliable to identify the calyceal fornix in nondilated collecting system under conventional US. The actual calyceal fornix was sometimes inconsistent with the imaging showed on conventional US and easy to be mistaken (Supplementary Fig. 3), which may account for the significant lower puncture accuracy in nondilated collecting systems. Retrograde US contrast injection can delineate morphological features of the collecting system, which is similar to iodinated contrast fluoroscopy. It helps to distinguish the renal calyx from peripelvic fat and locate the ideal calyceal fornix, thus improves the accuracy of puncture. We also noted that there were still five patients failed in puncture via calyceal fornix under CEUS guidance. It may be associated with lack of experience in the initial study phase, puncture needle deviation due to patient's respiratory activity and relative narrow calyx fornix in nondilated collecting systems. Nevertheless, our data proved that CEUS-guided PCNL facilitated more accurate puncture in patients with no or mild hydronephrosis, which accounted for lower postoperative hemoglobin loss and need of blood transfusion in CEUS-guided PCNL.

To the best of our knowledge, this is the first study to investigate the efficacy and safety of CEUS-guided PCNL in flank position. Currently, the most widely used position for patient undergoing PCNL is prone. However, it is not suitable for patients with cardiopulmonary disorders or skeletal deformities and may be associated with adverse outcomes in certain conditions [24]. Other positions such as flank and supine have been reported to be as effective and safe as prone position [25]. The advantages of flank position include less restriction of the respiratory movement of the chest wall, better ventilation to the endotracheal tube and adequate space for performing ultrasonography in multiple directions. Our results showed the stone-free rate and overall complication rate of CEUS-guided PCNL in flank position was comparable to that of PCNL performed in prone position for patients without hydronephrosis. Thus, the present study further extends the application of CEUS in PCNL and may benefit the patients who are not suitable for prone position.

There were several other advantages of CEUS-guided PCNL in the treatment of kidney stone patients with no apparent hydronephrosis. The efflux of urine through the puncture needle was sometimes difficult to observe even successful renal puncture was performed, especially in patients with unobvious artificial hydronephrosis. Under CEUS guidance, we could confirm the tip of the puncture needle enters the collection system after seeing a bright contrast outflow along with the needle, either by retrograde injection or antegrade injection of the contrast agent. Before the guide wire placement and dilation, we could also evaluate the puncture quality by observing the angle between the puncture needle and the axis of the renal calyx to the calyx neck. Another advantage of CEUS guidance is that multiple injections can be performed during one operation, owing to the short half-life (5–7 min) of US contrast, which was feasible for urologists to start this new technology. Last but not least, US contrast agent was safe and relative cheap, without significant effect on the complication rate of surgery or the economic burden of patients. These above-mentioned advantages of CEUS guidance may all contribute to a successful PCNL in kidney stone patients with no apparent hydronephrosis.

There were several limitations in the current study. First, this study represents a relatively small cohort reflecting a single institution’s initial experience. The clinical value of CEUS-guided PCNL needs to be validated in larger sample size study. Second, since this study was not blinded to the researchers, bias may exist in surgical interventions, data collection or analysis. Third, we only evaluated the feasibility of establishing single channel of minimally invasive PCNLs under CEUS guidance. Whether this technology is practical in multiple tracts or standard PCNL in flank position still needs further research. Lastly, all procedures were performed by two surgeons skilled in US-guided PCNL. The clinical value of CEUS in no or mild dilated collecting systems in flank position for trainees or novices still needs further investigation.

In conclusion, this study showed CEUS benefits more accurate renal puncture and less postoperative hemoglobin loss. CEUS-guided PCNL in flank position is a safe and effective approach for patients with no apparent hydronephrosis, and can be used as an alternative to fluoroscopy-guided PCNL without compromising outcomes.

## Supplementary Information

Below is the link to the electronic supplementary material.

Supplementary file1 The entry of the puncture needle into the collection system was confirmed by seeing a bright contrast outflow along with the needle (JPG 503 KB)

Supplementary file2 Postoperative intravenous pyelography shows puncture through renal calyx fornix (**A**) and puncture not through renal calyx fornix (**B**) (JPG 216 KB)

Supplementary file3 (JPG 376 KB)

Supplementary file4 The location of calyceal fornix showed on B-mode ultrasound (white arrow) was sometimes not the actual calyceal fornix (red arrow), which can be exactly identified under CEUS guidance (JPG 326 KB)

Supplementary file5 (DOCX 18 KB)

Supplementary file6 (DOCX 19 KB)

## Abbreviations

**CEUS**: Contrast-enhanced ultrasound
**PCNL**: Percutaneous nephrolithotomy
**US**: Ultrasound
