# Supplementary figures and images for: Feasibility of contrast-enhanced ultrasound and flank position during percutaneous nephrolithotomy in patients with no apparent hydronephrosis: a randomized controlled trial
Source: World J Urol. 2022 Jan 21;40(4):1043–8. doi: 10.1007/s00345-022-03933-4 (PMC8994732; doi:10.1007/s00345-022-03933-4)

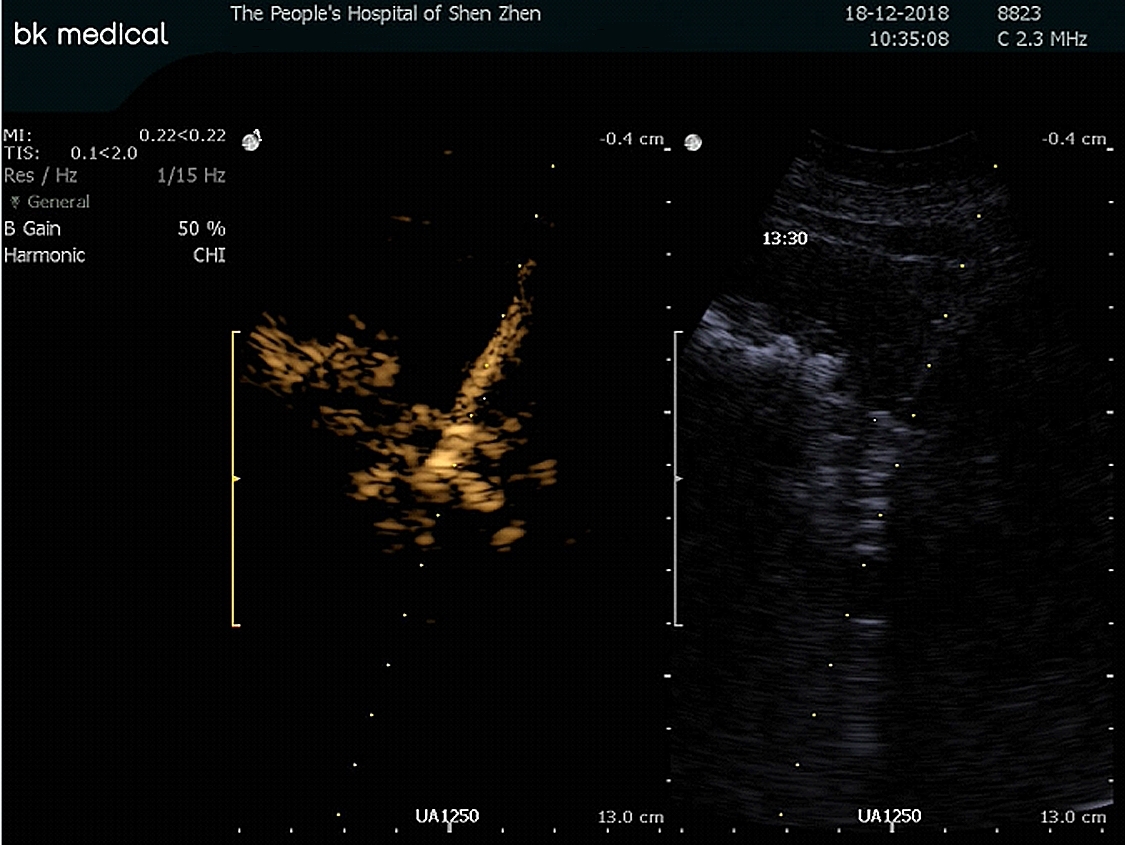

Supplement: Supplementary file 1 — Supplementary file1 The entry of the puncture needle into the collection system was confirmed by seeing a bright contrast outflow along with the needle (JPG 503 KB) [file 345_2022_3933_MOESM1_ESM.jpg]

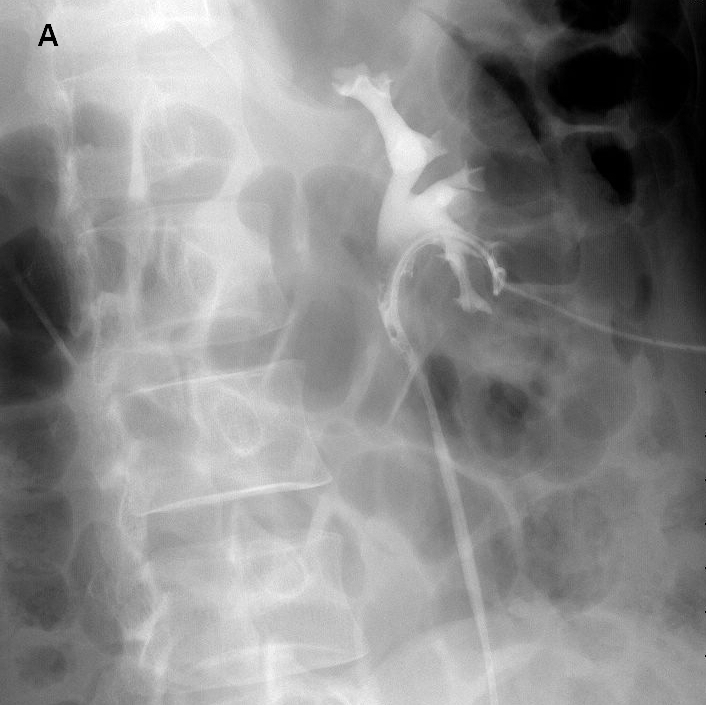

Supplement: Supplementary file 2 — Supplementary file2 Postoperative intravenous pyelography shows puncture through renal calyx fornix (A) and puncture not through renal calyx fornix (B) (JPG 216 KB) [file 345_2022_3933_MOESM2_ESM.jpg]

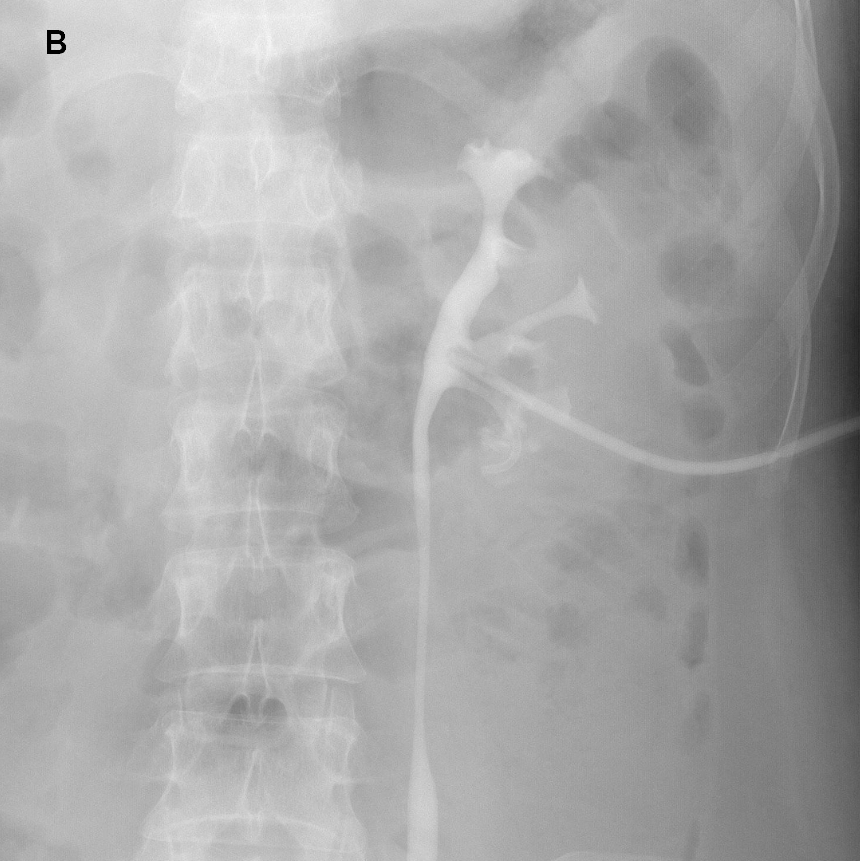

Supplement: Supplementary file 3 — Supplementary file3 (JPG 376 KB) [file 345_2022_3933_MOESM3_ESM.jpg]

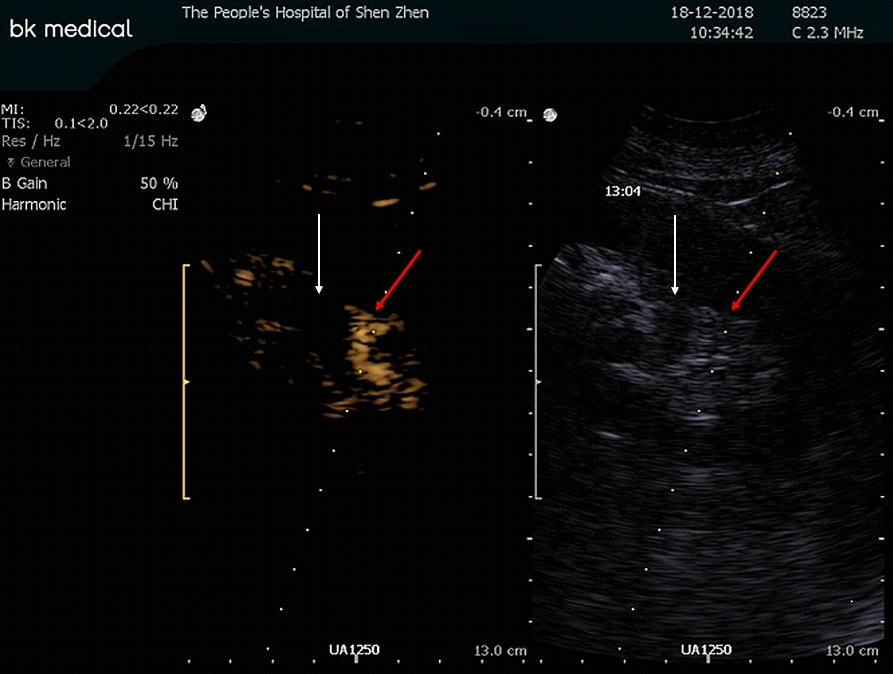

Supplement: Supplementary file 4 — Supplementary file4 The location of calyceal fornix showed on B-mode ultrasound (white arrow) was sometimes not the actual calyceal fornix (red arrow), which can be exactly identified under CEUS guidance (JPG 326 KB) [file 345_2022_3933_MOESM4_ESM.jpg]
