# Supplementary material for: Feasibility of contrast-enhanced ultrasound and flank position during percutaneous nephrolithotomy in patients with no apparent hydronephrosis: a randomized controlled trial
Source: World J Urol. 2022 Jan 21;40(4):1043–8. doi: 10.1007/s00345-022-03933-4 (PMC8994732; doi:10.1007/s00345-022-03933-4)
Supplement: Supplementary file 5 — Supplementary file5 (DOCX 18 KB) [file 345_2022_3933_MOESM5_ESM.docx]

**Table 1. Patients and renal stone characteristics**

| Variables | CEUS-guided PCNL (n=36) | US-guided  PCNL (n=36) | P |
| --- | --- | --- | --- |
| Age (years), mean (SD) | 45.9 (10.5) | 48.7 (10.4) | 0.27 |
| Gender, n (%) |  |  | 0.29 |
| Female | 8 (22.2%) | 12 (33.3%) |  |
| Male | 28 (77.8%) | 24 (66.7%) |  |
| BMI (kg/m^2^), mean (SD) | 23.8 (3.8) | 24.5 (3.3) | 0.42 |
| Stone size (mm), median (Q1, Q3) | 35.5 (24.0, 53.0) | 37.0 (25.5, 47.0) | 0.90 |
| Laterality, n (%) |  |  | 0.64 |
| Right | 17 (47.2%) | 19 (52.8%) |  |
| Left | 19 (52.8%) | 17 (47.2%) |  |
| Stone location, n (%) |  |  | 0.86 |
| Simple pelvic stone | 2 (5.6%) | 1 (2.8%) |  |
| Simple calyceal stone | 3 (8.3%) | 4 (11.1%) |  |
| Pelvis stone with calyceal stone | 10 (27.8%) | 10 (27.8%) |  |
| Partial staghorn stone | 8 (22.2%) | 11 (30.6%) |  |
| Complete staghorn stone | 13 (36.1%) | 10 (27.8%) |  |
| Degree of hydronephrosis |  |  | 1 |
| None | 21 (58.3%) | 21 (58.3%) |  |
| Mild | 15 (41.7%) | 15 (41.7%) |  |
