# Supplementary material for: Feasibility of contrast-enhanced ultrasound and flank position during percutaneous nephrolithotomy in patients with no apparent hydronephrosis: a randomized controlled trial
Source: World J Urol. 2022 Jan 21;40(4):1043–8. doi: 10.1007/s00345-022-03933-4 (PMC8994732; doi:10.1007/s00345-022-03933-4)
Supplement: Supplementary file 6 — Supplementary file6 (DOCX 19 KB) [file 345_2022_3933_MOESM6_ESM.docx]

**Table 2. Intraoperative and postoperative results**

| Variables | CEUS-guided PCNL (n=36) | US-guided  PCNL (n=36) | P |
| --- | --- | --- | --- |
| The number of successful puncture, n (%) | 36 (100%) | 33 (91.7%) | 0.24 |
| The number of attempts for successful puncture, n (%) |  |  | 0.10 |
| 1 | 33 (91.7%) | 28 (77.8%) |  |
| ≧2 | 3 (8.3%) | 8 (22.2%) |  |
| Puncture via calyceal fornix, n (%) | 31 (86.1%) | 17 (47.2%) | 0.002 |
| Puncture time (seconds),  median (Q1, Q3) | 36.5 (29.0, 46.8) | 61.0 (52.5, 76.5) | ＜0.001 |
| Operative time (minutes),  median (Q1, Q3) | 125.0 (97.3, 166.3) | 115.0 (95.0, 155.0) | 0.78 |
| Stone clearance rate, % (n) | 80.6% (29/36) | 60.6% (20/33) | 0.07 |
| Hemoglobin decrease (g/L),  median (Q1, Q3) | 2.5 (1.0, 10.0) | 14.5 (7.8, 18.3) | ＜0.01 |
| Postoperative stay (days),  median (Q1, Q3) | 6.0 (5.0, 6.0) | 6.0 (5.0, 8.0) | 0.40 |
| Postoperative complications, n (%) |  |  | 0.11 |
| Grade 0 | 30 (83.3%) | 31 (86.1%) |  |
| Grade 1 | 6 (16.7%) | 2 (5.6%) |  |
| Grade 2 | 0 (0.0%) | 3 (8.3%) |  |
| Grade 3 | 0 (0.0%) | 0 (0.0%) |  |
| Grade 4 | 0 (0.0%) | 0 (0.0%) |  |
